# The Potential Roles of Exosomes in Chronic Obstructive Pulmonary Disease

**DOI:** 10.3389/fmed.2020.618506

**Published:** 2021-01-14

**Authors:** Nan Wang, Qin Wang, Tiantian Du, Abakundana Nsenga Ariston Gabriel, Xue Wang, Li Sun, Xiaomeng Li, Kanghong Xu, Xinquan Jiang, Yi Zhang

**Affiliations:** ^1^School of Public Health, Shandong First Medical University and Shandong Academy of Medical Sciences, Taian, China; ^2^Department of Anesthesiology, Qilu Hospital, Shandong University, Jinan, China; ^3^Department of Clinical Laboratory, Cheeloo College of Medicine, The Second Hospital, Shandong University, Jinan, China; ^4^Department of Pharmacy, Binzhou Medical University Hospital, Binzhou, China; ^5^Respiratory and Critical Care Medicine Department, Qilu Hospital, Shandong University, Jinan, China

**Keywords:** exosomes, COPD, immune cell, biomarker, treatment

## Abstract

Currently, chronic obstructive pulmonary disease (COPD) is one of the most common chronic lung diseases. Chronic obstructive pulmonary disease is characterized by progressive loss of lung function due to chronic inflammatory responses in the lungs caused by repeated exposure to harmful environmental stimuli. Chronic obstructive pulmonary disease is a persistent disease, with an estimated 384 million people worldwide living with COPD. It is listed as the third leading cause of death. Exosomes contain various components, such as lipids, microRNAs (miRNAs), long non-coding RNAs(lncRNAs), and proteins. They are essential mediators of intercellular communication and can regulate the biological properties of target cells. With the deepening of exosome research, it is found that exosomes are strictly related to the occurrence and development of COPD. Therefore, this review aims to highlight the unique role of immune-cell-derived exosomes in disease through complex interactions and their potentials as potential biomarkers new types of COPD.

## Introduction

Chronic obstructive pulmonary disease (COPD) is a common chronic airway disease, characterized by irreversible, progressive airflow limitation, and repeated airway inflammation, which seriously affects the patient's breathing and interferes with the patient's life and work (1, 2). There are several viewpoints in COPD's pathogenesis, such as oxidative stress, epigenetics, cell aging, apoptosis, chronic inflammation, protease/antiprotease, and linear green body function (3). Environmental factors and genetic mutations play a role in it. Exposure to cigarette smoking(CS), whether active smoking or second-hand smoke, is the leading risk factor to get COPD (4, 5). Environmental pollution and occupational chemical exposure in some developing countries, are essential factors causing COPD (6). Exposure to indoor air pollution can affect unborn babies and is a risk factor for chronic obstructive pulmonary disease. According to the World Health Organization (WHO), COPD is the third leading cause of death globally. Statistical projections state that by 2040, the annual death toll will reach 4.4 million, and 90% of chronic obstructive pulmonary disease deaths will be more in both low-income and middle-income countries (7, 8). The airway epithelium CS events such as the first contact, long-term exposure to CS can induce epithelial cells to produce pro-inflammatory medium, senescence-associated secretory phenotype (MCP-1, IL-1, IL-6, IL-8), damage-associated molecular patterns (receptor for advanced glycation end-products, heat shock proteins, S100 proteins, high-mobility group box), the media which is released into the pulmonary and systemic circulation (9, 10), can stimulate the damage to the lung parenchyma, alveolar damage, and promote the development of COPD (11). Current treatment methods for COPD can only slow down the loss of lung function and cannot reverse the deterioration of lung structure and function. Therefore, it is essential to understand the molecular mechanism of COPD's occurrence and development and optimize the clinical treatment strategy. Various studies support the fact that exosomes containing miRNA, lncRNA, and proteins that are involved in the pathogenesis of COPD (12, 13), many exosomes, including miR-101, miR-223, miR-144, and miR-1274a act by influencing the molecular pathways associated with the pathogenesis of COPD which includes Kras, Notch, Smad, and TGF-β (14). Besides, these molecules are potential candidates for the early diagnosis and treatment of COPD. There are much-emerging shreds of evidence reporting that increased or decreased exosomes' expressions are a common feature of various lung diseases, including COPD (15). Takahashi et al. reported that changes in exosome numbers could predict a patient's physiological outcomes. For example, exosomes derived from bronchial endothelial cells were increased significantly in stable COPD (SCOPD), which were also increased in acute exacerbation COPD (AECOPD) (16).

Exosomes can maintain the stability of the intracellular environment (17). However, they can also get involved in different functions of the recipient cells (18). Direct exposure to a harmful stimulus (e.g., CS, air pollutants, and infection) may affect the nucleic acid load of lung-derived exosomes and participate in the progress of lung diseases (19). It was also found that exosomes may cause COPD pathological disturbances, that include chronic inflammation, oxidative stress, multiple organ dysfunction, epigenetic changes, cell apoptosis, aging, and diseases related to linear green body dysfunction (20, 21), and this proves that exosomes may act as a promising biomarker for the diagnosis of COPD (14). This is because, during the progression of COPD disease, COPD patients will produce a large number of exosomes, and can be isolated from sputum (22), plasma (23, 24), bronchial lavage fluid (BALF) (25), which suggests that exosomes considered as potential non-invasive diagnostic tools for COPD. This review first introduces the biological characteristics and functions of exosomes, introduces the role of exosomes in mediating intercellular communication and regulating immune cells in COPD, and finally clarifies the recent progress of exosomes in auxiliary clinical diagnosis and treatment of COPD.

## Main Components of Exosomes

### Biological Characteristics of Exosomes

Exosomes are membrane vesicles with a bilayer lipid of about 30–150 nm in diameter, produced by early and late endosomes, and eventually, form multivesicular bodies (MVB) (26). A variety of cells releases these vesicles, such as structure cells (epithelial cells, endothelial cells, and alveolar I and II, alveolar macrophages, fibroblasts), and immune cells (B and T lymphocytes, macrophages, dendritic cells) (3). CD9, CD63, CD81, and CD82 proteins from the peripheral membrane are fused during release into the extracellular space. These proteins are considered markers of exosomes (27). Besides, endosome markers also include heat shock 70 kDa protein 4 (Hsp70), MHC classes I and II, ALG-2 interacting protein X (Alix), and tumor susceptibility gene 101 (Tsg101) (28, 29).

### The Genetic Material in Exosomes

The main components of exosomes are protein and lipid, genetic material including DNA, mRNA, miRNA, lncRNA, and metabolites (30, 31). Among them, miRNA is a highly studied regulatory molecule. It has found that a small non-coding RNA between 19 and 25 nucleotides in length together with silencing the 3′-untranslated region (3′UTR) of the target mRNA, negatively regulates its expression at the transcriptional and post-transcriptional levels, which in return affects various cellular functions such as proliferation, apoptosis, differentiation, and emergency resistance. The events mentioned above also affect biological processes that include autophagy, inflammatory response, cellular senescence, tissue remodeling, immune regulation, and angiogenesis (32, 33). These processes usually involve the level of multiple genes. Besides, the length of the lncRNA is over 200 nucleotides, and located in the nucleus, transcripted by RNA polymerase II, but cannot make reaction with proteins; lncRNA has been shown to participate in mRNA splicing after transcription and protein translation process, usually expresses the tissue specificity and cell specificity, and in the maintenance of steady-state (34). More information about these two types of RNA is summarized in Table 1.

**Table 1:** miRNA vs. lncRNA.

	**miRNA**	**lncRNA**
Source	Pri-miRNA	Multiple sources
Length	19–25 nt	>200 nt
Protein coding	no	no
Regulatory target	mRNA 3′UTR	Proteins, RNAs, and DNAs
Effect	Up-regulate or downregulate mRNA	Up-regulate or downregulate target
Mechanisms	Inhibits gene expression at the transcriptional and post-transcriptional levels	Maintain the dynamic balance of cells and tissues and regulate inflammation

*miRNA, microRNA; lncRNA, long non-coding RNAs*.

### Biological Functions of Exosomes

**Figure 1:**
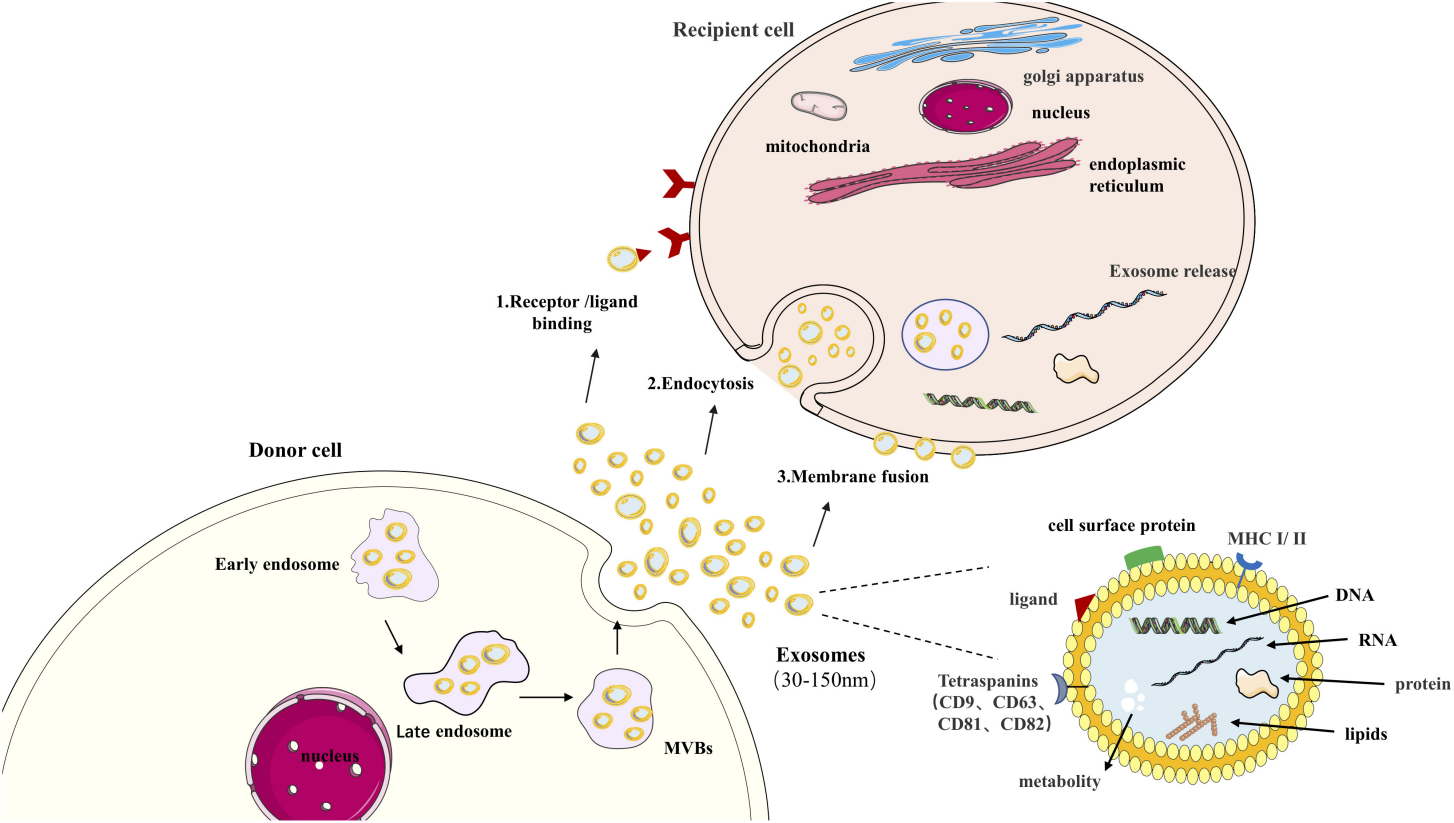
Exosome in cell communication. Exosomes are nanovesicles (30–150 nm) that originate formed by early endosomes, late endosomes, and ultimately multivesicular bodies (MVB). Exosomes contain a variety of proteins, lipids, DNA, RNA, and metabolites. The cell body's surface includes major histocompatibility complex (MHC I/II), Tetraspanins (CD9, CD63, CD81, CD82), cell surface proteins ligand. This confers them with functional features. Exosomes can transmit information to target cells by: (1) binding to the cell surface by ligand, (2) endocytosis, (3) direct fusion of extracellular with plasma membranes.

Exosomes can transmit information to target cells in three ways: (1) Interact with the cell surface through receptors; (2) Endocytosis; (3) The direct fusion of outer cell membrane and plasma membrane as it is shown in Figure 1. Briefly, exosomes' primary function is to transport essential substances and mediate communication between cells (35). This biological effect unit regulates the properties of target cells (36, 37). More evidence shows that exosomes are involved in different respiratory diseases, including COPD (38). In airway physiology, exosomes from alveolar macrophages, epithelial cells, and endothelial cells affect the disease's progress in controlling the stability of the intracellular environment and changing the function of receptor cells (39). Under normal circumstances, exosomes are highly stable in biological fluids due to the bilayer protection of lipids. High-throughput sequencing technology can detect highly sensitive and highly specific exosomes. A large amount of exosome secretion can be detected in various biological fluids, such as sputum, blood, BALF, urine, pleural effusion, ascites, synovial fluid, and breast milk (40). This makes exosomes potentially valuable for the diagnosis, prognosis, and treatment of respiratory diseases.

## The Clinically Relevant Roles of Exosomes in COPD

**Figure 2:**
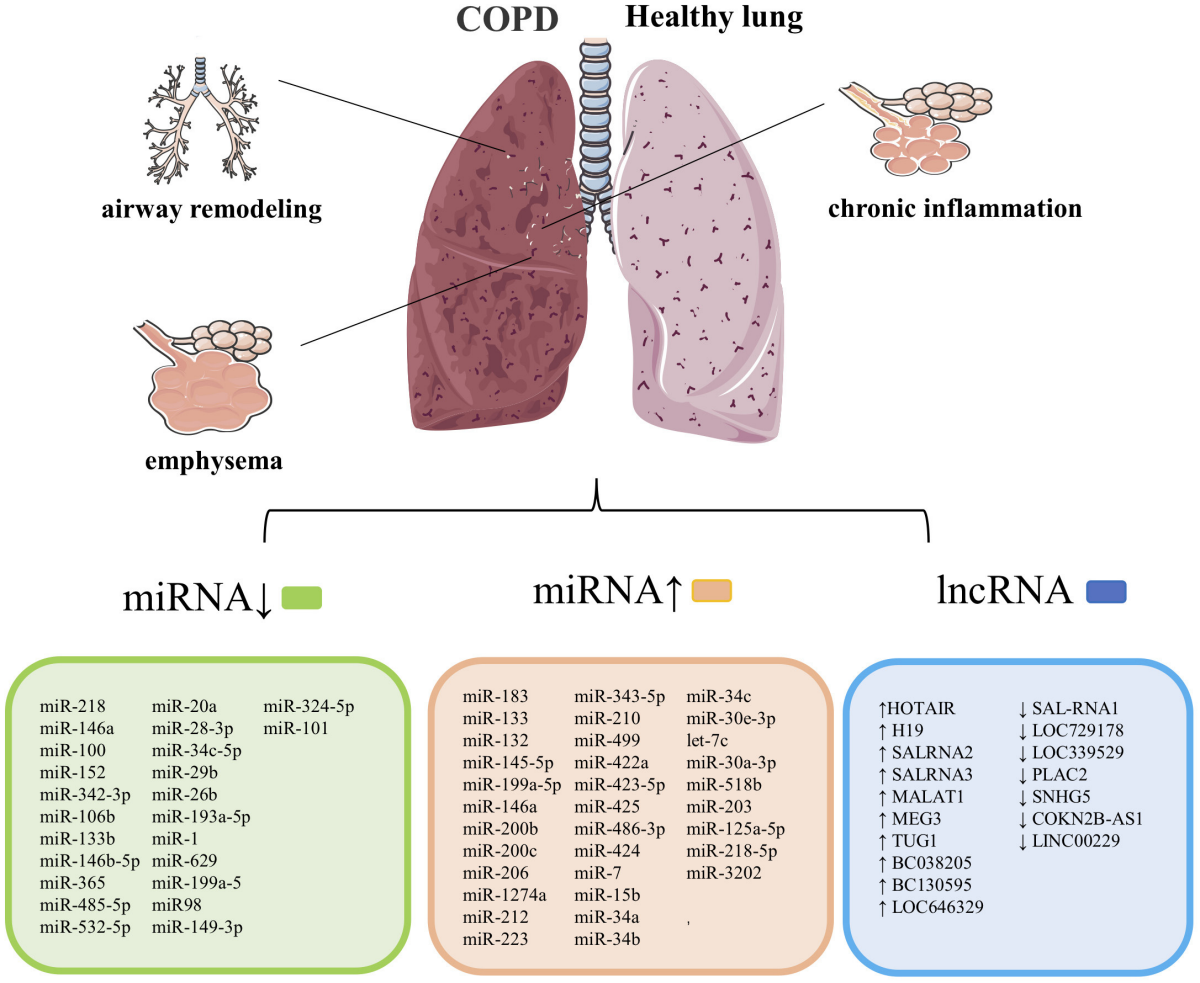
COPD's pathological features are chronic inflammation of the lung parenchyma and surrounding airways, emphysema, and narrowing. Remodeling of the small airways, and exosomes are closely related to the course of this cases. List of previously reported miRNA associated with COPD. COPD, chronic obstructive pulmonary disease. Summary data are based on the following references (4, 14, 15, 42–52) for miRNAs and (53–56) for lncRNAs.

Chronic obstructive pulmonary disease is a chronic inflammatory respiratory disease characterized by irreversible airflow limitations caused by prolonged exposure to cigarette smoke or harmful irritants. Multiple studies have demonstrated that exosomes play an essential role in the occurrence and progression of COPD, including inflammation of the lung parenchyma and surrounding small airways (chronic bronchitis), degeneration of lung tissue (emphysema), and remodeling of small airways, leading to decreased lung function (41). The related exosomes miRNAs and lncRNAs involved in COPD's development and progression are summarized in Figure 2.

### The Role of Exosomes in the Pathogenesis of COPD

#### Exosomes and Chronic Airway Inflammation

Cordazzo et al. reported that cigarette smoke extract (CSE) could induce exosomes to be released from mononuclear cells, induce bronchial epithelial cells to produce IL8, monocyte chemoattractant protein-1, and up-regulate CD542, thereby promoting inflammation (57). Also, Tan et al. believe that inflammation and exosomes increase in patients with COPD Relevant, they used Exo ELISA kits to compare the levels of CD9^+^ exosomes in COPD patients and healthy controls, and found that the levels of COPD patients were significantly higher (58). Moon et al. believe that exosomes rich in COPD-related protein cysteine-rich angiogenic protein 61 (CCN1) can be cleaved after CS exposure. CCN1 allows inflammatory signaling to the far end of the lung. Interaction with integrin 7 in pulmonary epithelial cells activates the secretion of matrix metalloproteinase protein (MMP-1) in pulmonary epithelial cells, thereby promoting the release of vascular endothelial growth factor and IL-8.Il-8 promotes neutrophil infiltration into the lung parenchyma and causes inflammation (59). Besides, after CSE treatment, MEG3 up-regulated in 16HBE cells of COPD patients can release the expressions of inflammatory factors IL1, IL6, and TNF by regulating HSA-Mir-218, thus inducing inflammation and cell apoptosis.

#### Exosomes and Emphysema

Emphysema is characterized by decreased airway elasticity, hyperinflation, and swelling of the terminal bronchioles (60). Ezzie et al. believe that transforming growth factor β (TGF-β) suggested changes in miRNA expression, such as miR-223 and miR-15b. After knocking out the transforming growth factor TGF R-II in mouse lung epithelial cells, they showed emphysema signs (14). Van Pottelberge et al. analyzed miRNA expression in sputum samples from 32 subjects using qPCR. The results showed that the expression of miR-125b and let-7C in PATIENTS with COPD was lower than that in healthy non-smokers. Low let-7C expression was negatively correlated with the concentration of tumor necrosis factor receptor 2(TNFR2) (42).

Furthermore, Stockley and Turne reported that glycoproteins alpha one anti-trypsin (A1AT) works by inhibiting the process of preventing excessive inflammation neutrophils and eosinophil enzymes work, and A1AT deficiency will lead to the development of emphysema (61). It was also found that in the evaluation of miRNA expression in lung tissue of patients with COPD patients with mild and moderate emphysema, moderate five miRNAs in lung samples of emphysema patients were significantly down-regulated, namely miR-34c, miR34b, miR-149, miR-133a, and miR-133b. Among them, the expression of miR-34c was significantly down-regulated (62).

#### Exosomes and Airway Remodeling

Long-term harmful stimuli lead to damage to airway epithelial cells, promote phenotypic changes of epithelial cells, and lead to fibrosis and airway remodeling. Fujita's research showed that CS-induced down-regulation of miR-210 could drive the differentiation of myofibroblasts in airway remodeling and inhibit the expression of ATG7 in LFS to control the autophagy process directly (63). This indicates that exosomes may contribute to the tracking of COPD differentiated fibers. miR-15b may be involved in airway remodeling by mediating TGF- related signaling pathways (15). Besides, it has also revealed that different microRNAs such as miR-224, miR-339-5p, and miR-382 are involved in COPD's pathogenesis (64), and particular pieces of information are presented in Table 2.

**Table 2:** List of miRNAs potentially associated with COPD pathogenesis.

**miRNA**	**Expression**	**Target**	**Function/pathogenesis**	**References**
miR-210	↑	ATG7	Promoted myofibroblast differentiation in primary lung fibroblasts control autophagy	(63)
miR-144	↑	CFTR, IRF7	Regulate inflammatory and antiviral responses in COPD	(43)
miR-101	↑	CFTR, MKP1		
miR-223	↑	TGF-β	TGF-β signal activation is associated with emphysema	(14)
miR-15b	↑	SMAD7	TGF-β inhibitory primary intracellular signal smad2/3/4	(14)
miR-218	↓	NF-κB	Up-regulates the expression of several pro-inflammatory cytokines by AEC, including IL-6 and CXCL8.	(65)
miR-128-5p	↓	apoptotic processes	Participate in the epithelial damage and remodeling in COPD	(44)
miR-181d	↑	and growth factor pathways		(43)
miR-1274a	↑	FOXO4	Probably related to oxidative stress or cellular aging in COPD	(14)
miR-1	↓	AKT	COPD-associated skeletal muscle dysfunction	(66)
miR-542-5p	↑	–		(45)
miR-542-3p				

*↑Upregulation; ↓downregulation*.

Gu's research shows that taurine-upregulated gene 1 (TUG1) expression is enhanced in COPD patients, and TUG1 inhibits the expression of miR-145-5pp/DUSP6 axis to promote airway remodelings such as TGF-β1, mucus hypersecretion, collagen 1 and α-SMA and inflammation such as increased IL-6 and pulmonary neutrophil infiltration (67). TUG1, combined with CDKN2B-AS1, can be used to predict acute exacerbation in COPD patients (68). It was found that the expressions of SAL-RNA2 and SAL-RNA3 (senescence-related genes) were significantly increased in COPD lung tissues, and the expressions of p53 and P21 were up-regulated. This shows that lncRNA in airway epithelial cells II type error control in the process of aging may be involved in the pathogenesis of COPD (53). In summary, exosomes influence COPD's occurrence and development by regulating inflammatory, emphysema, airway remodeling, and pulmonary fibrosis. MiRNAs and lncRNAs involved in the development of COPD are summarized in Figure 2.

### Immune Cell-Derived Exosome Participate in COPD

Exosomes are recognized as tools for mediating intercellular communication and signal transduction and signaling to immune cells to regulate function. Various studies highlighted that exosomes are secreted by some types of cells, including immune cells, such as B lymphocytes (69), T lymphocytes (70), macrophages (Mϕ) (71), mast cells (MC) (30), dendritic cells (DC) (72), and 17 natural killer cells (NK) (73). Immune-cell-derived exosomes perform numerous physiological and pathological functions, including antigen presentation, immunosuppression, T-cell polarization toward Treg cells, and anti-inflammatory effects, to enhance or suppress immune activity (74). Infiltration of various immune cells, such as neutrophils and macrophages, into small airways, is a common phenomenon in COPD (75). For these reasons, in-depth research is needed to explore the role of immune-cell-derived exosomes in COPD.

#### Role of Macrophages and Exosomes in COPD

**Figure 3:**
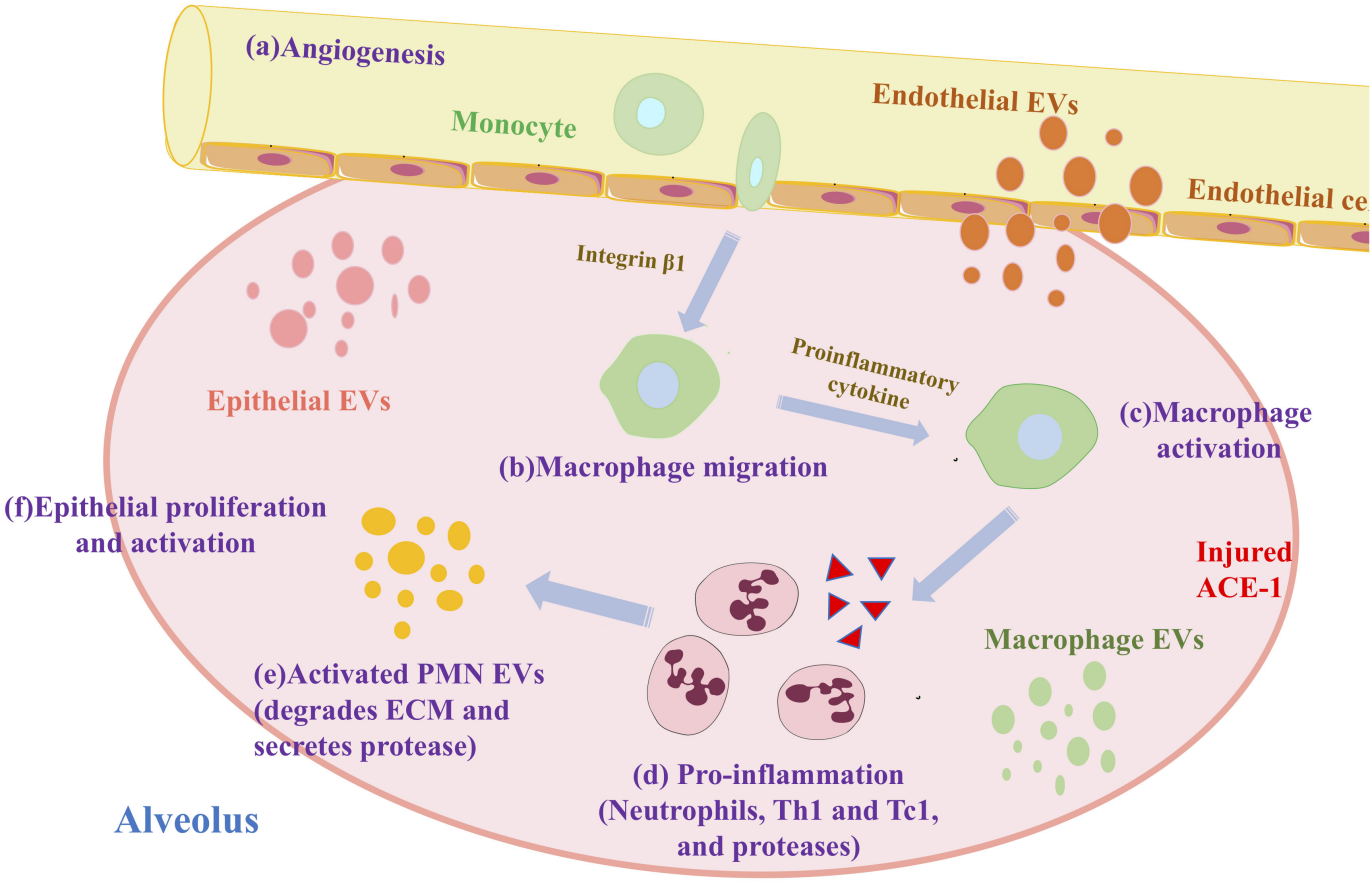
Exosomes and macrophages promote alveolar inflammation in COPD.

Alveolar macrophages (AM) form the first host defense line against infection/inhalation of harmful substances. They are one of the primary cells in the lung that produce exosomes (76). AM promotes inflammation of COPD. First, macrophage-derived exosomes induce differentiation of immature monocytes into macrophages by mediating miR-223 (77). Exosomes miR-17 and miR-221 then promote macrophage migration by mediating integrin β under sterility stimulation (78). Finally, exosomes miR-221 and miR-320 stimulate the secretion of pro-inflammatory cytokines, thereby activating AM (79). After a series of differentiation, migration, and activation, AM recruits neutrophils, Th1, and Tc1, to promote inflammation (32). The specific process is shown in Figure 3.

After CS stimulation, miRNA mediated by endothelial cells, such as miR-125a, miR-126, miR-191, were transferred to macrophages to promote apoptotic cell clearance (80). A study reported that macrophages release outside secretes body with antigen-presenting ability lower associated macrophage transfer specific immune (81). Kulshreshtha et al. found that epithelial-derived exosomes extracted from BALF of mice with COPD induced undifferentiated macrophages and proliferative effects (82). During the pathogenesis of COPD, alveolar macrophages combine with suppressor of cytokine signaling (SOCS) protein, which is taken up by alveolar epithelial cells and inhibits IFN γ-induced signal transducer and activator of transcription (STAT) (58). Also, M1 macrophages (pro-inflammatory) are the predominant cells in the peripheral blood of COPD patients, leading to pro-inflammatory factors (IL-1β, IL-12, TNF-α) and lncRNA miR-155HG are up-regulated (83). As mentioned above, the studies show that the increase of exosomes in the regulation of lung inflammation by macrophages in the lung has practical significance.

#### Role of Neutrophils and Exosomes in COPD

Neutrophils are a crucial component of innate immune responses in COPD, and Kristopher described a new pathogen, activated PMN (neutrophil) source of NE-rich exosomes (84). These exosomes bind and degrade the extracellular matrix (ECM) involved in COPD's pathogenesis. Mac-1 and neutrophilic elastase (NE) play essential roles in this process. NE is the surface binding protease obtained by CD63^+^/CD66b^+^ nanovesicles during PMN degranulation, and NE is resistant to A1AT. It is because of the resistance of the A1AT. The ECM targeting, the outer body of the outer body, which is more potent than the free NE biology (85), and an imbalance of the proteinase-antiprotease system are typical in COPD (86). In this system, PMN-derived NE and lung suppressor A1AT are most prominent (87). Experiments have shown that injecting enough NE into an animal's airways can cause emphysema (88). These findings show that novel exosomes derived from neutrophils participate in disease progression by breaking ECM dynamic balance through NE activity in COPD.

#### Role of Other Cells and Exosomes in COPD

Among mast cells, the exosomes of human mast cell line-1 overlapped with the proteins of the exosomes inhaled by the patient, suggesting that mast cells also contribute to the lung exosome pool (89). It is worth noting that exosomes from NK cells can react against NK cells and increase the cytotoxic activity and NK cells' targeting (90). Besides, exosomes released by macrophages, NK cells, and dendritic cells through a paracrine act as pro-inflammatory mediators of the innate immune system (81). Exosomes secreted by DC are associated with amplification of the immune response, and DC-based exosomes induce specific humoral responses and activation of CD4^+^T cells and CD8^+^T cells (91). As mentioned above, immune cell-derived exosomes are involved in the development and progression of COPD. Exosomes derived from circulating immune cells have immunomodulatory effects. Therefore, it has been reported that immune-cell-derived exosomes can be delivered to target cells by small molecule drugs or specific molecules to stimulate the immune system in patients to recognize and destroy lesions (90). Also, exosomes derived from circulating immune cells can serve as specific biomarkers for the severity of inflammation, which has been demonstrated in liver inflammation (92, 93). The application of exosomes derived from immune cells in immunotherapy is now considered a promising tool in COPD treatment. In conclusion, there are still many questions to be answered about the relationship between immune cells and exosomes involved in lung inflammation and injury in COPD.

## Clinical Application of Exosomes in COPD

### Exosomes as Potential Biomarkers for the Clinical Diagnosis of COPD

The study results compared healthy exosomes and exosomes released by bronchial epithelial cells in COPD patients, especially in the AECOPD, and the study reported that exosomes from AECOPD were significantly increased. Moreover, they found that by detecting exosomes' content, pulmonary endothelial cell apoptosis can be analyzed. The degree of activation and the dysfunction of endothelial cells is directly related to the severity of COPD. It is usually evaluated by forced expiratory volume in 1 s (FEV1) to track the disease's advancement (16). Lacedonia and his research team reported that the number of exosomes in sputum is negatively correlated with FEV1, similar to changes in exosome levels related to inflammation. It shows that senescent cells accumulated with age in COPD may affect the release and competition of circulating exosomes (94). Moreover, Shi et al. reported that miR-203 might be a new biomarker for COPD diagnosis; it is just because miR-203 targets P13KCA to inhibit nuclear factors light chain enhancers, which activate B cell signaling pathways significantly involved in COPD (95). Another critical study showed that evidence suggests that miR-21 has potential value in the diagnosis and treatment of COPD. The level of miR-21 in the serum of smokers and patients with COPD is significantly increased. The increased level of miR-21 is associated with the percentage of forced expiratory volume in 1 s/forced vital capacity (FEV1/FVC) (18).

Besides, comparing miR-4455 and miR-4785 in smokers and non-smokers in COPD patients, there was a significant expression difference (96). This made the two exosomal microRNAs to be considered as a potential biomarker for early diagnosis of COPD. Chronic obstructive pulmonary disease, early diagnosis, and patient monitoring are other essential factors. Based on that, researchers conducted a study. They found that miR-218-5p is another essential miRNA that plays an essential role in COPD pathogenesis and promises to be a successful candidate for diagnosing and controlling COPD patients (65). Moreover, other essential study findings showed that miR-29c and miR-126 were up-regulated in Stage III COPD patients relative to stable controls. Such results indicate that all these miRNAs may be used as screening biomarkers for COPD patients (97). As mentioned above from different studies, the findings show that exosomal miRNAs can be used as potential biomarkers for the diagnosis of COPD.

### Application of Exosomes in COPD Prognosis

Studies have shown that exosomes with CD144, CD31, and CD62E are more abundant in patients with AECOPD than in patients with SCOPD, suggesting that COPD patients tend to deteriorate can be used as an indicator of prognosis (16). Ge et al. detected the expression of ANRIL in SCOPD patients, AECOPD, and the whole group. They found that the expression of ANRIL in AECOPD patients was the lowest. The expression of ANRIL in SCOPD patients was not related to the GOLD stage. In contrast, the expression of ANRIL in patients with AECOPD was negatively correlated with the GOLD stage. Most importantly, ANRIL expression in patients with AECOPD and stable COPD is negatively correlated with inflammatory cytokines such as IL1B, IL17A, TNF, and LTB4 (98). They finally concluded that lncRNA ANRIL (antisense non-coding RNA at INK4 locus) is associated with chronic diseases' occurrence and prognosis.

It is well-known that skeletal muscle weakness is a severe systemic complication of COPD, which seriously affects patients' quality of life and mobility. Lewis et al. showed that exosome miR-1 plays a crucial role in skeletal muscle dysfunction in patients with COPD, associated with smoking history, function, defatted body mass index, 6-min walking distance, and percentage of quadriceps type I muscle fibers (66). Besides, compared with healthy COPD patients with fat-free body mass index (FFMI), H19 is expressed by demethylation isolation of has-miR-519a after up-regulation increases patients' susceptibility with COPD to low FFMI (99). So far, researchers have used exosomes as a potential non-invasive biomarker for COPD that has never stopped. In short, these studies are mainly used to identify the role of exosomes in the diagnosis of COPD, providing a new basis for the early clinical diagnosis of some exosomes in COPD. Through continuous efforts, new diagnostic tools and more complete treatment methods may appear.

### Exosomes Are Essential in the Treatment of COPD

#### COPD Treatment Using Exosomal miRNA

At present, the drugs used to treat COPD are mainly bronchodilators, which are borrowed from medications used to treat asthma. Inhalation corticosteroids (ICSs) are added to LAMA/LABA combination therapy (triple therapy), and the risk of exacerbation and death in COPD patients. However, most patients are not sensitive to steroids, and the use of large doses increases the risk of pneumonia (100). Therefore, COPD patients urgently need critical target treatment. There is also a great need for drugs to prevent the aggravation of the disease and pulmonary hypertension, plus many other COPD complications.

Sun's study reported that miR-206 expression in human pulmonary microvascular endothelial cells (HPMECs) exposed to CS was up-regulated. They also noted that both Caspase3 and HPMEC apoptosis activities were increased. There was a negative correlation between the expression levels of miR-206 and Notch3 and VEGFA mRNA levels. During their study, they concluded that miR-206 could directly target Notch3 and VEGF-A to regulate the process of COPD vascular remodeling (101). It was reported that anti-miR-27-3p could effectively reverse the inflammatory process and reduce the infiltration of neutrophils and macrophages in the lungs and the level of inflammatory cytokines in BALF. Another study aimed to determine miRNA-3202 in COPD noted that the overexpression of miR-3202 could significantly inhibit the increase of T lymphocyte IFN-γ and TNF-α levels induced by CSE while increasing the expression of Fas and FasL. The study also revealed that the high levels of miR-3202 pass target gene Fas apoptosis inhibitory molecule (FAIM) 2 to inhibit T cell apoptosis and protect human bronchial epithelial cells (HBECs). FAIM2 plays a central role in COPD's pathogenesis, inhibits T cell death, and participates in CS-induced cell apoptosis and cell wall destruction (64). Besides, it has also been found that the high levels of miR-145-5p alleviate CS-induced apoptosis and the production of pro-inflammatory cytokines by inhibiting the lytic expression of p53 and caspase339. Besides, some miR-145-5p and KLF5 inhibits CSE-induced NF-κB signal activation, thereby reducing participation in the pathogenesis of COPD (102).

Du et al. tested the expression rates of miR-181c in 34 COPD (smoking cases) patients relative to safety controls, and findings showed that miR-181c might be slightly less controlled in COPD patients than healthy control patients had never smoked. They also demonstrated that the upregulation of miR-181c might be correlated with several consequences, such as inflammatory response reduction, neutrophil invasion, reactive oxygen species formation, and inflammatory cytokine development. MiR-181c downregulation may be correlated with different consequences. They also found that miR-181c exerts its impact by attacking CCN1. The downregulation of the miR-181c could contribute to the increase in CCN1 expression in the pulmonary tissues of patients with COPD relative to stable controls. The results mentioned above show that miR-181c might be used as a therapeutic target to treat COPD patients (103). Another study also reported that the deregulation of miR-126 is correlated with ATM kinase activation, and they have also demonstrated that miR-126 levels have been decreased in the smoker and COPD endothelial blood cells relative to non-smoker subjects. These findings indicated that the reduction of miR-126 through ATM targeting might facilitate tissue aging and dysfunction in smokers and COPD subjects. This miRNA may also be seen as a new therapeutic target for treating COPD patients (104). Taken together, it is clear that exosomes play an essential role in the treatment of COPD patients.

#### The Treatment of COPD by Synthetic siRNA

Synthetic siRNAs' potential to treat COPD by transcriptionally down-regulating target genes' expression has been successfully validated in multiple animal models (105). For example, mitogen-activated protein kinase kinase kinase (MAP3K) 19 phosphorylates the nucleus of Smad2/3 Translocation and activation of NF-κB reduces lung neutrophil infiltration and BALF levels of KC (a mouse homolog of human IL-8) (106). A recent study has shown that it is caused by infection of the upper respiratory tract. Among the causes of worsening COPD, human rhinovirus (HRV) and non-typeable Hemophilus influenza (NTHI) is the most common. The combined HRV/NTHI response increased IL-17C production, and IL-17C-specific siRNA could block IL-17C and CXCL1 and neutrophil migration induced by HRV/NTHI (107). Trophoblast cells' surface antigen (Trop) 2 expressed on the basal lung compartment's multipotent progenitor cells in preventing COPD airway remodeling Surface antigen. Its specific siRNA reverses cell movement and migration of basal cell hyperplasia. Epithelial-mesenchymal transition (EMT) (108), COPD combined pulmonary hypertension is a common complication of patients, which is closely related to the high expression of the neuron-derived orphan receptor (NOR)1.NOR1 plays a role in regulating inflammation and vascular remodeling. Its specific siRNA inhibits hypoxia-induced cyclin D1 level, cell proliferation, and DNA synthesis in primary human pulmonary artery smooth muscle cells (PASMCs) (109) SI00A4, a secretory member of S100 calcium-binding protein, is highly expressed in COPD patients and mouse models and is involved in the proliferation, migration, and EMT of smooth muscle cells. HIF-1α or HIF-2α siRNA can inhibit the high expression of S100A4 (110). Based on the above facts, exosomes play a crucial role in the treatment of COPD. However, COPD is a disease caused by a variety of factors.

## Future Direction and the Limitation

The study of exosomes vs. COPD is an emerging and rapidly evolving field where we can use exosomes to develop therapeutic tools to prevent and stop lung injury caused by COPD. However, there are still many problems to be solved, such as the lack of uniformity and standardization of exosome detection, isolation, and purification technologies. The advantages and disadvantages of exosome isolation methods are shown in Table 3. Secondly, the mechanism of exosome uptake by recipient cells is not fully understood. Also, collecting tissue samples from the lungs is more complicated. More in-depth studies are needed to fully understand the pathogenesis of exosomes in COPD and fully reveal the role of miRNA and lncRNA in the disease.

**Table 3:** Principle, advantages and disadvantages of common exosome isolation methods.

**Methods**	**Theory**	**Advantages**	**Disadvantages**	**References**
Ultracentrifugation techniques (UC)	The required components are obtained according to the size and density differences of each component in the sample.	There is no need to mark the outer cut body to avoid cross contamination	High cost, Time consuming, structural failure, aggregation and lipoprotein separation are not conducive to downstream analysis	(46)
Density gradient centrifugation	Usually used in combination with the overspeed centrifuge method	Improve the purity of exosomes	The high viscosity of sucrose solution will reduce the settling velocity of exosomes and lead to long time consuming	(47)
Polymer Precipitatio	Using the method of virus extraction, exosomes is obtained by reducing the solubility of exosomes, usually using polyethylene glycol (PEG) as medium	Simple operation, short analysis time, suitable for large—dose sample treatment	Due to its low purity and recovery rate, the resulting polymer is difficult to remove and may produce false positive	(48)
Size-based isolation techniques	Based on the size differences between exosomes and other components of a biological sample	Fast, simple, low-cost and separated exosomes have complete structure and uniform size, and their biological characteristics will not be significantly affected.	Other particles of similar size are difficult to separate, resulting in reduced purity	(46)
Ultrafiltration	Ultrafiltration membranes with different molecular weight cutoffs (MWCO) were used to selectively separate the samples	The sample cost is low, the concentration efficiency is high, and the activity of the exosomes is not affected	Low purity and poor binding of the exosomes to the ultrafiltration membrane resulted in low recovery rate.	(49)
Immunoaffinity chromatography (IAC)	The specificity of antibodies and ligand is combined to separate the required exosomes from heterogeneous mixtures	The sample size required is small.It can be used for the qualitative and quantitative detection of exosomes.This method has strong specificity, high sensitivity, high purity and high yield	The preservation condition of the exosomes obtained by this method is harsh, and it is not suitable for large scale separation of the exosomes. The non-specific interference adsorption of matrix produces interfering proteins, which limits the wide application of this method.	(50)

## Conclusion

Chronic obstructive pulmonary disease is a heterogeneous disorder marked by airway inflammation, lung tissue damage, and airflow restriction consistent with airway remodeling. Exacerbations are a significant cause of disease development, morbidity, and death, and novel therapies and medications for COPD remain particularly crucial due to a lack of effective drugs. Exosomes are incredibly durable and practical packages of cellular material. They can transfer their bioactive loads to receiver cells across the body, influencing physiological and pathological conditions. Exosomes have considerable clinical value due to their capacity to be controlled and optimized to guide treatment. Although this review tried to highlight the latest evidence of exosomes from various cell sources as biomarkers and their potential for application in COPD treatment, in-depth researches are needed to be used in daily early diagnosis and treatment of COPD.

## Author Contributions

NW and QW designed and drafted the manuscript. TD, AG, XW, LS, XL, and KX discussed and revised the manuscript. All authors read and approved the final manuscript.

## Conflict of Interest

The authors declare that the research was conducted in the absence of any commercial or financial relationships that could be construed as a potential conflict of interest.

## Abbreviations

AECOPD: acute exacerbation chronic obstructive pulmonary disease
A1AT: alpha one anti-trypsin
Alix: ALG-2 interacting protein X
AM: alveolar macrophages
BALF: bronchial lavage fluid
CAMP: cyclic adenosine monophosphate
CCN1: protein cysteine-rich angiogenic protein 61
CCR10: chemokine receptor 10
COPD: chronic obstructive pulmonary disease
COX2: cyclooxygenase-2
CS: cigarette smoking
CSE: cigarette smoke extract
DC: dendritic cells
ECM: extracellular matrix
EMT: epithelial-mesenchymal transition
FAIM: Fas apoptosis inhibitory molecule Hsp70: heat shock 70 kDa protein 4
FEV1: forced expiratory volume in 1 s
FEV1/FVC: forced expiratory volume in 1 s/forced vital capacity
FFMI: fat-free body mass index
HBECs: human bronchial epithelial cells
HPMECs: human pulmonary microvascular endothelial cells
HRV: human rhinovirus
ICSs: Inhalation corticosteroids
lncRNA: long non-coding RNA
MAP3K: mitogen-activated protein kinase kinase kinase
MC: mast cells
miRNA: microRNA
MMP: matrix metalloproteinase protein
MVB: multivesicular bodies
Mϕ: macrophages
NE: neutrophilic elastase
NK:17: natural killer cells
NTHI: non-typeable Hemophilus influenza
NOR: neuron-derived orphan receptor
PASMCs: pulmonary artery smooth muscle cells
SCOPD: stable chronic obstructive pulmonary disease
SOCS: suppressor of cytokine signaling
siRNA: Small interfering RNA
STAT: signal transducer and activator of transcription
TGF-β: transforming growth factor β
TNFR2: tumor necrosis factor receptor 2
Trop: Trophoblast cells surface antigen
Tsg101: tumor susceptibility gene 101
TUG1: taurine-upregulated gene 1
UCHL1: ubiquitin C-terminal hydrolase
WHO: World Health Organization.
